# Kefir Supplementation Modifies Gut Microbiota Composition, Reduces Physical Fatigue, and Improves Exercise Performance in Mice

**DOI:** 10.3390/nu10070862

**Published:** 2018-07-04

**Authors:** Yi-Ju Hsu, Wen-Ching Huang, Jin-Seng Lin, Yi-Ming Chen, Shang-Tse Ho, Chi-Chang Huang, Yu-Tang Tung

**Affiliations:** 1 Graduate Institute of Sports Science, National Taiwan Sport University, Taoyuan 33301, Taiwan; 1041302@ntsu.edu.tw (Y.-J.H.); 1021302@ntsu.edu.tw (Y.-M.C.); 2 Department of Exercise and Health Science, National Taipei University of Nursing and Health Sciences, Taipei 11219, Taiwan; magicpica521@gmail.com; 3 Culture Collection & Research Institute, SynbioTech Incorporation, Kaohsiung 821, Taiwan; jslin@synbiotech.com.tw; 4 Health Technology Collage, Jilin Sport University, Changchun 130022, Jilin, China; 5 Department of Animal Science and Technology, National Taiwan University, Taipei 10673, Taiwan; stho1129@ntu.edu.tw; 6 Graduate Institute of Metabolism and Obesity Sciences, Taipei Medical University, Taipei 11031, Taiwan; 7 Nutrition Research Center, Taipei Medical University Hospital, Taipei 11031, Taiwan

**Keywords:** kefir, gut microbiota, antifatigue, exercise performance

## Abstract

The present study evaluated the potential beneficial effect of kefir (KF) against fatigue. Furthermore, the composition of the gut microbiota is related to health benefits in the host; therefore, the study also investigated the effect of KF on the gut microbiota composition. Male ICR mice from four groups (*n* = 8 per group) were orally administered KF once daily for four weeks at 0, 2.15, 4.31, and 10.76 g/kg/day and were designated as the vehicle, KF-1X, KF-2X, and KF-5X groups, respectively. The gut microbiota was analyzed using 16S rRNA gene sequencing. The results showed a significant clustering of cecum after treatment in the vehicle, KF-1X, KF-2X, and KF-5X groups. The KF-2X and KF-5X groups showed a decreased *Firmicutes*/*Bacteroidetes* ratio compared with the vehicle group. In addition, anti-fatigue activity and exercise performance were evaluated on the basis of exhaustive swimming time, forelimb grip strength, and levels of serum lactate, ammonia, glucose, blood urea nitrogen (BUN), and creatine kinase (CK) after a swimming exercise. The exhaustive swimming time for the KF-1X, KF-2X, and KF-5X groups was significantly longer than that for the vehicle group, and the forelimb grip strength of the KF-1X, KF-2X, and KF-5X groups was also significantly higher than that of the vehicle group. KF supplementation also decreased serum lactate, ammonia, BUN, and CK levels after the swimming test. However, tissue glycogen content, an important energy source for exercise, increased significantly with KF supplementation. Thus, KF supplementation can alter the gut microbiota composition, improve performance, and combat physical fatigue.

## 1. Introduction

The health-promoting effect of fermented dairy are the result of the bioactive ingredients present in native milk and, also, due to their suitably modulated activities produced by the action of probiotic bacteria, in the fermented or sour milk products [1]. The health benefits of fermented dairy materialize either by direct interaction with consumed microorganisms or indirectly due to the effects of microbial metabolites produced during the fermentation process [1]. Kefir (KF), which originates from the Caucasus Mountains, is an acidic fermented milk with trace amounts of alcohol [2]. KF is traditionally produced by inoculating milk with a relatively stable and specific KF grain that contains lactic acid bacteria and yeast. The beverage has become an important functional dairy product, and in the past decade, research into KF has increased [3]. KF has been used for the clinical treatment of hypertension, gastrointestinal diseases, allergies, and ischemic heart disease [2,4]. In addition, KF possesses many biological activities, including antibacterial, antifungal, antimutagenic, antioxidant, antidiabetic, antitumor, and immune-stimulating effects; it is also effective against fatty liver syndrome [5]. Moreover, KF consumption provides beneficial bacteria, yeast, vitamins, minerals, fatty, and complete protein. Recent research showed that dairy lipids possess anti-chronic inflammation bioactivity [6]. In addition, recent research and meta-analyses showed full-fat dairy consumption has higher bioavailability of high-value nutrients and anti-inflammatory properties [7]. Thus, kefir is a healthy nutrient-rich food that is beneficial for the anti-inflammation and immune system, and has been used as a supplement for patients with AIDS, chronic fatigue syndrome (CFS), herpes, and cancer [8].

During exercise, many energy sources (e.g., glucose and glycogen) are exhausted, resulting in physical fatigue [9]. Some biomarkers including lactate, ammonia, blood urea nitrogen (BUN), and glucose are widely used to evaluate fatigue [10,11]. Regular exercise can improve body functions, but strenuous exercise can lead to the accumulation of reactive oxygen species and lipid peroxides, thereby damaging the organs and causing fatigue [12,13]. Therefore, fatigue is a matter of concern because it can further lead to various disorders in relation to bio-regulatory, autonomic nervous, endocrine, and immune system. These disorders related to biological regulation and the immune system can also lead to reduced exercise intensity and disruption of daily activities [14].

Fatigue is the common physiological reactions and its symptoms are tiredness and lack of energy. Long-term fatigue is easily causing aging, human immunodeficiency virus (HIV) infection, depression, Parkinson’s disease, multiple sclerosis, and cancers [15]. Therefore, many researchers are interested in the use of kefir, herbal medicines, natural compounds, dietary foods, or exercise equipment technology to delay fatigue and accelerate the elimination of fatigue-related metabolites [16,17,18]. The fermented milk product was effective in reducing the impact of exercise-induced immune suppression [19]. O’Brien et al. [18] showed consumption of kefir has the positive health benefits on performance and recovery after exercise during intensive endurance training. Changes in the gut microbiota composition have been reported to cause chronic fatigue syndrome/myalgic encephalomyelitis (CFS/ME) [20]. Logan et al. [21] reported that low levels of bifidobacteria and gut bacterial overgrowth can lead to immune dysfunction in ME/CFS patients. Sheedy et al. [22] observed a significant increase in the proportion of lactic acid produced by *Enterococcus* and *Streptococcus* in the fecal samples of ME/CFS patients. However, relatively few studies directly involve anti-fatigue activity of KF and its effect on the gut microbiota composition. Therefore, in the current study, we used our established in vivo platform [23,24] to evaluate anti-fatigue effects and analyze gut microbiota composition after KF supplementation.

## 2. Materials and Methods

### 2.1. KF Preparation

KF was obtained from SYNBIO TECH INC. (Kaohsiung, Taiwan). It was prepared by inoculating pasteurized 9.2% reconstituted skim milk with powder kefir starter culture and fermented at 37 °C for 16 h. The fermented milk was then pasteurized at 100 °C for 30 min and freeze dried. The powder kefir starter culture used for inoculation was composed of defined lactic acid bacteria strains which contains *Lactobacillus fermentum* DSM 32784 (LF26), *L. helveticus* DSM 32787 (LH43), *L. paracasei* DSM 32785 (LPC12), *L. rhamnosus* DSM 32786 (LRH10), and *Streptococcus thermophilus* DSM 32788 (ST30). All strains were isolated from traditional kefir. A 100-g portion of KF contains 354.75 calories with 30 g of protein, 0.75 g of fat, and 57 g of carbohydrates. The samples were initially stored in airtight containers at 4 °C until further use.

### 2.2. Animals and Experiment Design

Male ICR mice (age, six weeks; weight, 25 g) were purchased from BioLASCO (A Charles River Licensee Corp., Yi-Lan, Taiwan). The mice were given rodent chow 5001 and distilled water ad libitum. They were housed at room temperature (24 ± 2 °C) under a humidity-controlled (65 ± 5%) condition on a regular 12-h light/dark cycle. Animal protocol was reviewed and approved by the Institutional Animal Care and Use Committee (IACUC) of National Taiwan Sport University, Taoyuan City, Taiwan (IACUC-10523).

After a two-week acclimation period, the ICR mice (age, eight weeks) were divided into four groups based on body weight (*n* = 8 per group): (1) the vehicle control group (vehicle group); (2) supplementation with KF-1X group (KF-1X group); (3) supplementation with KF-2X group (KF-2X group); and (4) supplementation with KF-5X group (KF-5X group). The KF administered to the KF-1X, KF-2X, and KF-5X groups was 2.15, 4.31, and 10.76 g/kg/day, respectively. The vehicle group was administered glucose water with the same calorie content as the KF administered to the KF-1X, KF-2X, and KF-5X groups. The volume of glucose water or supplement administered to all groups was the same, and the dose was determined according to the body weight of each mouse. The KF preparation or glucose water was orally administered once daily for 28 days.

### 2.3. Exhaustive Swimming Test

Results of the exhaustive swimming tests were determined according to the method of Kan et al. [25]. Results of the exhaustive swimming test were determined 30 min after KF supplementation on day 29. The swimming time from beginning to exhaustion was used to evaluate endurance performance.

### 2.4. Forelimb Grip Strength

A low-force testing system (Model-RX-5, Aikoh Engineering, Nagoya, Japan) was used to measure the forelimb absolute grip strength, as previously described [16]. Grip strength was measured 30 min after KF supplementation on day 28.

### 2.5. Fatigue-Associated Biochemical Indices

On day 31, blood samples were collected after 10 min of the swimming exercise and after 20 min of rest. Serum was centrifuged for 10 min (1500× *g*) at 4 °C. Lactate, ammonia, and glucose levels were determined using an autoanalyzer (Hitachi 7060, Hitachi, Tokyo, Japan). After 33 days of the intervention, the mice were subjected to a 90-min swimming test after 60 min of rest to evaluate fatigue-associated changes in CK and BUN levels.

### 2.6. Tissue Glycogen Determination

At the end of the experiments on day 36, the glycogen contents of liver and muscles were analyzed. The method of the glycogen analysis was assayed according to a previously described method [16].

### 2.7. Histological Staining of Tissues

Different tissues were collected and fixed in 10% formalin after the mice were sacrificed. The hematoxylin and eosin staining according to a previously described method by Huang et al. [10].

### 2.8. Blood Biochemical Assessments

At the end of the experiments on day 36, all mice were fasted for 8 h, sacrificed using 95% CO_2_ asphyxiation, and had blood was withdrawn through cardiac puncture. Serum was collected through centrifugation, and the levels of aspartate aminotransferase (AST), alanine aminotransferase (ALT), albumin, creatinine, lactate dehydrogenase (LDH), CK, total protein (TP), glucose, total cholesterol (TC), and triacylglycerol (TG) were assessed using an autoanalyzer (Hitachi 7060, Hitachi, Tokyo, Japan).

### 2.9. Bacterial DNA Extraction and 16S rRNA Sequencing

Cecum samples were collected on day 36. The collected samples were immediately stored at −80 °C for DNA extraction. The bacterial DNA was extracted using the cetyltrimethylammonium bromide/sodium dodecyl sulfate (CTAB/SDS) method and directly used in polymerase chain reaction (PCR) assays and 16S rRNA gene sequencing. DNA concentration and purity were monitored on 1% agarose gels. The extracted DNA was stored at −80 °C prior to 16S rRNA sequencing. The hypervariable V3–V4 region of the bacterial 16S rRNA gene was amplified using PCR with bar-coded universal primers 341F (F, forward primer; 5′-CCTAYGGGRBGCASCAG-3′) and 806R (R, reverse primer; 5′-GGACTACNNGGGTATCTAAT-3′). Library construction and sequencing of amplicon DNA samples were performed using BIOTOOLS Co., Ltd. (New Taipei City, Taiwan). A pair-end library (insert size of 450–470 bp for each sample) was constructed using the TruSeq DNA PCR-Free Sample Preparation Kit (Illumina, San Diego, CA, USA), and high-throughput sequencing was performed on an Illumina HiSeq2500 platform.

### 2.10. Statistical Analysis

Experimental data are expressed as mean ± SD (*n* = 8). A one-way analysis of variance (ANOVA) was employed to calculate the significance differences between multiple groups with Duncan’s test, and *p* values of <0.05 were considered significant. The Cochran–Armitage trend test was examined the dose effect.

## 3. Results

### 3.1. Effect of Four-Week KF Supplementation on Tissue Weights, Body Weight, Food Intake, and Water Intake

Table 1 showed body weight, food intake, and water intake. The muscle and brown adipose tissue (BAT) mass of the mice in the KF-1X, KF-2X, and KF-5X groups was higher than that in the vehicle group. These results indicated no significant differences in the weights of the liver, kidney, epididymal fat pad (EFP), heart, and lung tissues, or in the food and water intake among the vehicle, KF-1X, KF-2X, and KF-5X groups.

### 3.2. Effect of Four-Week KF Supplementation on the Exhaustive Swimming Test

**Figure 1:**
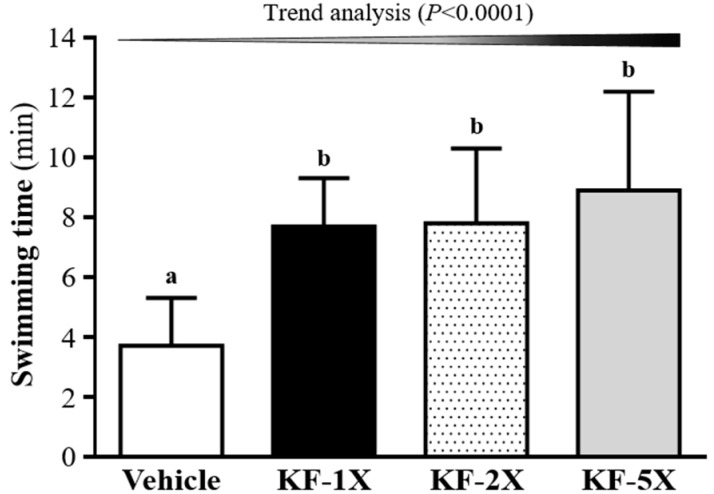
Effects of kefir (KF) supplementation on the exhaustive swimming test. Data are presented as mean ± SD, *n* = 8. Bars with different letters (^a^, ^b^) indicate a significant difference at *p* < 0.05 determined using one-way ANOVA. Vehicle (glucose water), KF-1X (2.15 g/kg/day KF), KF-2X (4.31 g/kg/day KF), and KF-5X (10.76 g/kg/day KF).

As shown in Figure 1, the exhaustive swimming time of the KF-1X group was 7.7 ± 1.6 min (2.10-fold longer than that of the vehicle group; *p* = 0.0019); the exhaustive swimming time of the KF-2X group was 7.8 ± 2.5 min (2.12-fold longer than that of the vehicle group; *p* = 0.0017); and the exhaustive swimming time of the KF-5X group was 8.9 ± 3.3 min (2.43-fold longer than that of the vehicle group; *p* = 0.0001), indicating that the KF-1X, KF-2X, and KF-5X groups exhibited an anti-fatigue effect. Furthermore, a significant dose-dependent effect on endurance swimming performance was observed (*p* < 0.0001).

### 3.3. Effect of Four-Week KF Supplementation on the Forelimb Grip Strength

**Figure 2:**
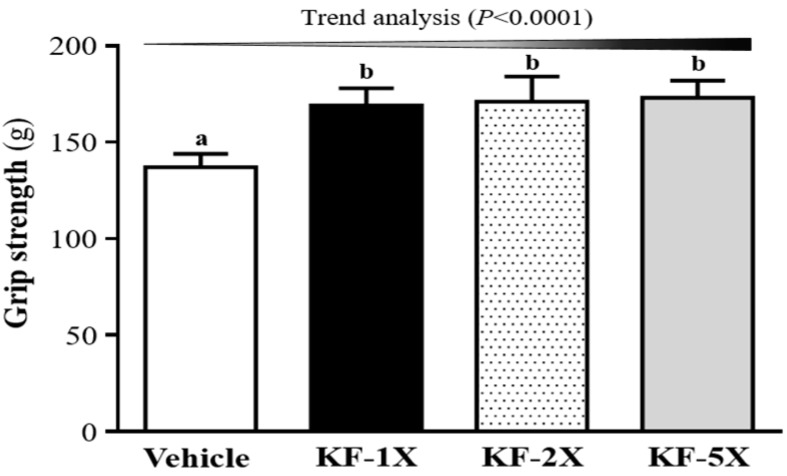
Effects of kefir (KF) supplementation on the forelimb grip strength. Data are presented as mean ± SD, *n* = 8. Bars with different letters (^a^, ^b^) indicate a significant difference at *p* < 0.05 determined using one-way ANOVA. Vehicle (glucose water), KF-1X (2.15 g/kg/day KF), KF-2X (4.31 g/kg/day KF), and KF-5X (10.76 g/kg/day KF).

Grip strength was higher in the KF-1X (169 ± 9 g), KF-2X (171 ± 13 g), and KF-5X (173 ± 9 g) groups than in the vehicle group (137 ± 7 g) (*p* < 0.001) (Figure 2). Thus, the grip strength in the KF-1X, KF-2X, and KF-5X groups significantly increased by 23%, 25%, and 26%, respectively, compared with that in the vehicle group. In addition, a significant dose-dependent effect on the grip strength was observed (*p* < 0.0001).

### 3.4. Effect of Four-Week KF Supplementation on Lactate after the 10-Min Swimming Test

**Figure 3:**
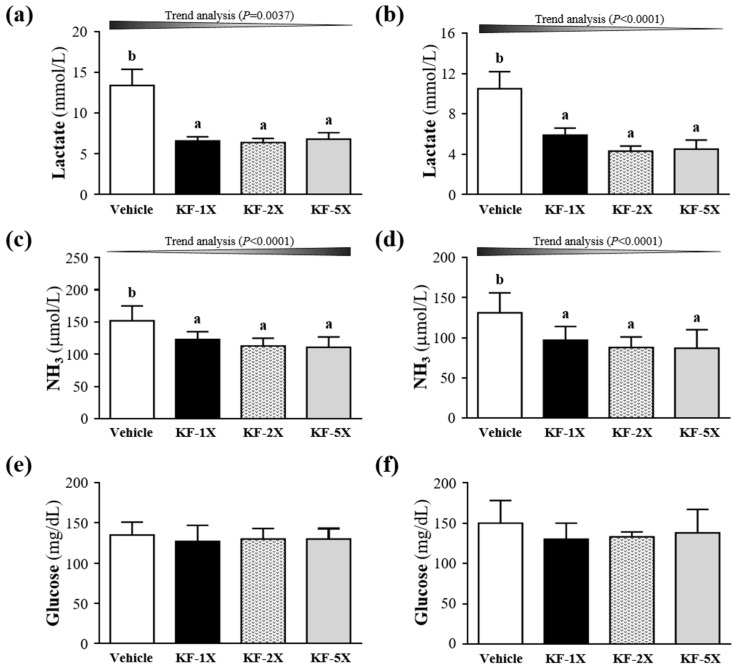
Effects of kefir (KF) supplementation on the serum levels of (**a**,**b**) lactate, (**c**,**d**) ammonia, and (**e**,**f**) glucose after a 10-min swimming test (**a**,**c**,**e**) and after a 20-min resting period (**b**,**d**,**f**). Data are presented as mean ± SD, *n* = 8. Bars with different letters (^a^, ^b^) indicate a significant difference at *p* < 0.05 determined using one-way ANOVA. Vehicle (glucose water), KF-1X (2.15 g/kg/day KF), KF-2X (4.31 g/kg/day KF), and KF-5X (10.76 g/kg/day KF).

On day 31 of the intervention, the mice were subjected to a 10-min swimming test to evaluate lactate levels before and after the swimming exercise and after 20 min of rest (Figure 3a). Before swimming, no significant differences were observed in blood lactate levels among the vehicle (3.0 ± 0.2 mmol/L), KF-1X (2.9 ± 0.3 mmol/L), KF-2X (2.9 ± 0.1 mmol/L), and KF-5X (3.0 ± 0.2 mmol/L) groups. After swimming, the blood lactate levels in the KF-1X (6.6 ± 0.5 mmol/L), KF-2X (6.4 ± 0.5 mmol/L), and KF-5X (6.8 ± 0.8 mmol/L) groups were significantly lower (50.5%, *p* < 0.0001; 52.4%, *p* < 0.0001; and 49.0%, *p* < 0.0001, respectively) compared with that in the vehicle group (13.4 ± 2.0 mmol/L). In addition, a significant dose-dependent effect on blood lactate levels was observed (*p* = 0.0037) after the 10-min swimming test. During the 20-min rest period, the blood lactate levels in the KF-1X (5.9 ± 0.7 mmol/L), KF-2X (4.3 ± 0.5 mmol/L), and KF-5X (4.5 ± 0.9 mmol/L) groups were significantly lower (43.6%, *p* < 0.0001; 59.4%, *p* < 0.0001; and 57.1%, *p* < 0.0001, respectively) than that in the vehicle group (10.5 ± 1.7 mmol/L) (Figure 3b). A significant dose-dependent effect on blood lactate levels was also noted (*p* < 0.0001) after the 10-min swimming test and 20-min rest period.

### 3.5. Effect of Four-Week KF Supplementation on Ammonia and Glucose after the 10-Min Swimming Test

After 31 days of the KF supplementation, the serum ammonia levels in the KF-1X (123 ± 12 μmol/L), KF-2X (113 ± 12 μmol/L), and KF-5X (111 ± 16 μmol/L) groups were 19.0% (*p* = 0.0016), 25.7% (*p* < 0.0001), and 27.3% (*p* < 0.0001), respectively, lower than that in the vehicle group (152 ± 23 μmol/L) after the swimming test (Figure 3c). Moreover, a significant dose-dependent effect on blood ammonia levels was observed (*p* < 0.0001) after the 10-min swimming test. During the 20-min rest period, the blood ammonia levels in the KF-1X (97 ± 17 μmol/L), KF-2X (88 ± 13 μmol/L), and KF-5X (87 ± 23 μmol/L) groups were significantly lower (26.0%, *p* = 0.0019; 33.0%, *p* = 0.0002; and 33.9%, *p* = 0.0001, respectively) than that in the vehicle group (131 ± 25 μmol/L) (Figure 3d). Moreover, a significant dose-dependent effect on blood ammonia levels (*p* < 0.0001) was also noted after the 10-min swimming test and 20-min rest period.

### 3.6. Effect of Four-Week KF Supplementation on BUN and CK after the 90-Min Swimming Test and 60-Min Rest Period

**Figure 4:**
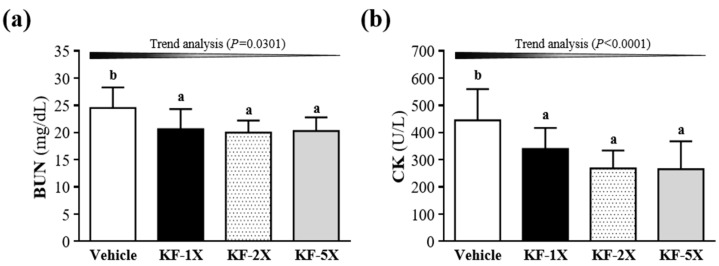
Effects of kefir (KF) supplementation on the serum levels of (**a**) blood urea nitrogen (BUN) and (**b**) creatine kinase (CK) after a 90-min swimming test and a 60-min resting period. Data are presented as mean ± SD, *n* = 8. Bars with different letters (^a^, ^b^) indicate a significant difference at *p* < 0.05 determined using one-way ANOVA. Vehicle (glucose water), KF-1X (2.15 g/kg/day KF), KF-2X (4.31 g/kg/day KF), and KF-5X (10.76 g/kg/day KF).

The BUN levels in the KF-1X (20.6 ± 3.7 mg/dL), KF-2X (20.0 ± 2.2 mg/dL), and KF-5X (20.3 ± 2.5 mg/dL) groups were significantly lower (16.1%, *p* = 0.0170; 18.3%, *p* = 0.0074; and 17.3%, *p* = 0.0110, respectively) than that in the vehicle group (24.5 ± 3.8 mg/dL) (Figure 3a). Moreover, a significant dose-dependent effect on the BUN levels was observed (*p* = 0.0301). As shown in Figure 4b, the serum CK levels in the KF-1X (339 ± 78 U/L), KF-2X (268 ± 66 U/L), and KF-5X (265 ± 103 U/L) groups were significantly lower (23.8%, *p* = 0.0297; 39.7%, *p* = 0.0007; and 40.5%, *p* = 0.0006, respectively) compared with that in the vehicle group (445 ± 115 U/L). In addition, a significant dose-dependent effect on the CK levels was observed (*p* < 0.0001).

### 3.7. Effect of Four-Week KF Supplementation on Liver and Muscular Glycogen

**Figure 5:**
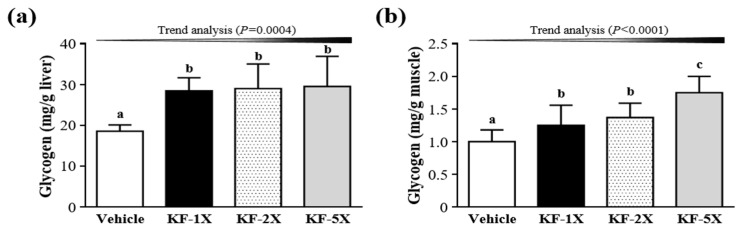
Effects of kefir (KF) supplementation on (**a**) liver glycogen and (**b**) muscular glycogen. Data are presented as mean ± SD, *n* = 8. Bars with different letters (^a^, ^b^, ^c^) indicate a significant difference at *p* < 0.05 determined using one-way ANOVA. Vehicle (glucose water), KF-1X (2.15 g/kg/day KF), KF-2X (4.31 g/kg/day KF), and KF-5X (10.76 g/kg/day KF).

As shown in Figure 5a, liver glycogen contents in the KF-1X (28.44 ± 3.25 mg/g liver), KF-2X (29.00 ± 6.06 mg/g liver), and KF-5X (29.54 ± 7.38 mg/g liver) groups were significantly higher (1.53-fold, *p* = 0.0006; 1.56-fold, *p* = 0.0003; and 1.59-fold, *p* = 0.0002, respectively) than that in the vehicle group (18.58 ± 1.53 mg/g liver). In addition, a significant dose-dependent effect on liver glycogen content was observed (*p* = 0.0004). However, muscular glycogen levels in the KF-1X (1.25 ± 0.31 mg/g muscle), KF-2X (1.37 ± 0.22 mg/g muscle), and KF-5X (1.75 ± 0.25 mg/g muscle) groups were higher (1.25-fold, *p* = 0.0489; 1.37-fold, *p* = 0.0051; and 1.74-fold, *p* < 0.0001, respectively) than those in the vehicle group (1.00 ± 0.18 mg/g muscle) (Figure 5b). Moreover, a significant dose-dependent effect on muscle glycogen content was observed (*p* < 0.0001).

### 3.8. Effect of Four-Week KF Supplementation on Histopathology of Tissues and Biochemical Variables at the End of the Experiment

**Figure 6:**
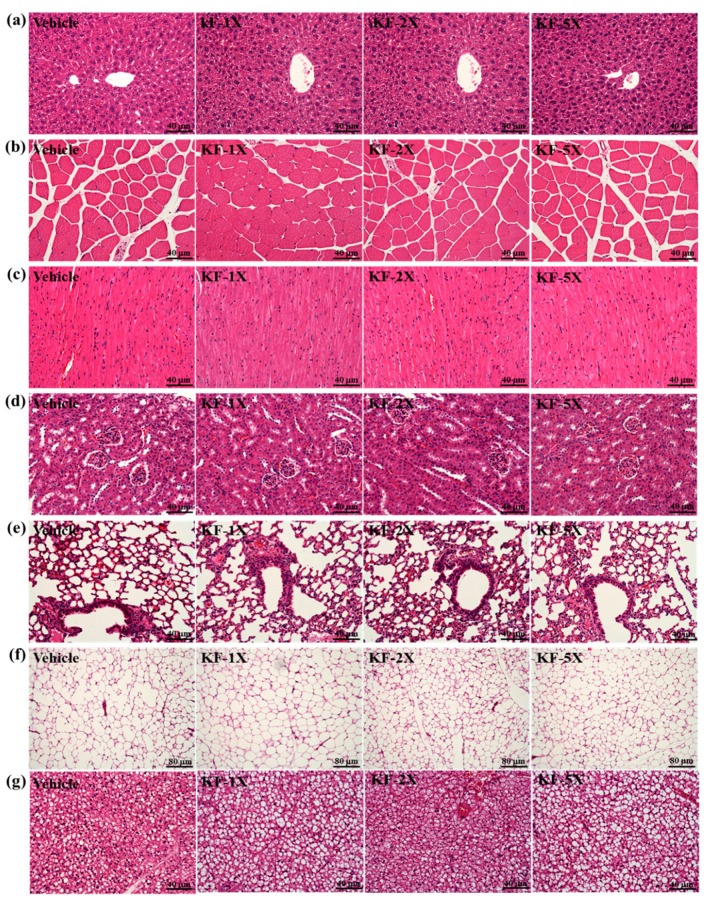
Effects of kefir (KF) supplementation on (**a**) liver; (**b**) muscle; (**c**) heart; (**d**) kidney; (**e**) lungs; (**f**) epididymal fat pad (EFP); and (**g**) brown adipose tissue (BAT). Specimens were observed using a light microscopy. Hematoxylin and eosin stain, magnification: ×200 (**a**–**e**) and ×100 (**f**,**g**). Vehicle (glucose water), KF-1X (2.15 g/kg/day KF), KF-2X (4.31 g/kg/day KF), and KF-5X (10.76 g/kg/day KF).

The pathological histology of the major organs, including the liver, muscle, heart, kidney, and lung tissues, is shown in Figure 6. The histological observations of the liver, muscle, heart, kidney, lungs, EFP, and BAT of the mice in the KF-1X, KF-2X, and KF-5X groups did not differ from those in the vehicle group. No clinical signs of organ-specific toxicity were observed after KF treatments. The ALT and CK levels of the mice in the KF-1X, KF-2X, and KF-5X groups were lower than those in the mice from the vehicle group. Other biochemical indices, including AST, ALT, albumin, creatinine, LDH, CK, TP, glucose, TC, and TG, did not differ among the four groups (Table 2). Therefore, the doses of KF supplementation used in the present study were safe.

### 3.9. Effect of Four-Week KF Supplementation on the Gut Microbiota

**Figure 7:**
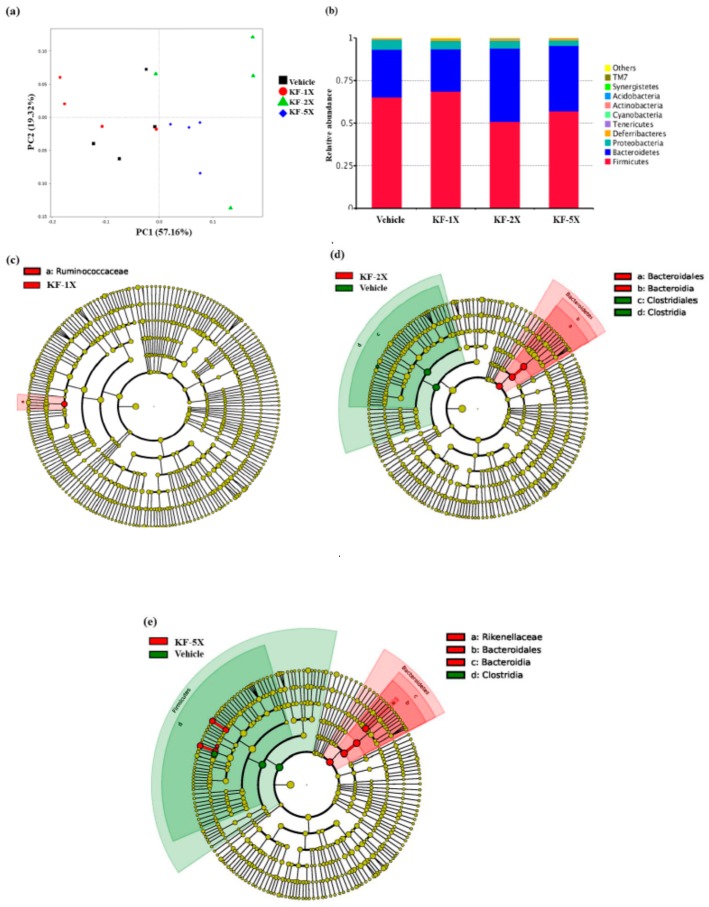
(**a**) Effects of kefir (KF) supplementation on principal coordinate analysis (PCoA) of the gut microbiota composition in mice based on the Bray–Curtis distance measure of samples in the relative abundance profiles of an operational taxonomic unit; (**b**) Effects of KF supplementation on the phylum-level gut microbiota composition of mice. Only phyla with top 10 average abundances were included, with other phyla collapsed into “Others”; the effects of KF supplementation on the phylum-level gut microbiota composition of mice in the (**c**) KF-1X and vehicle groups; (**d**) KF-2X and vehicle groups, and (**e**) KF-5X and vehicle groups. The cladogram was generated from the linear discriminant analysis effect size (LEfSe) analysis, showing the most differentially abundant taxa enriched in the microbiota of mice in (**c**) the KF-1X (red) and vehicle (green) groups, (**d**) the KF-2X (red) and vehicle (green) groups, and (**e**) the KF-5X (red) and vehicle (green) groups. Significant differential abundances in LEfSe comparisons are sorted by *p* values in ascending order: vehicle (glucose water), KF-1X (2.15 g/kg/day KF), KF-2X (4.31 g/kg/day KF), and KF-5X (10.76 g/kg/day KF).

We analyzed the gut microbiota composition using the 16S rRNA genes in the KF-1X-, KF-2X-, and KF-5X-treated mice and observed dramatic changes in the microbial ecology when treated with KF for 36 days. A principal coordinate analysis showed that mice clustered into relatively distinct groups based on different treatments (Figure 7a), thus suggesting that KF significantly altered the gut microbial populations. Figure 7b indicates that, at the phylum level, the overall composition of the gut microbiome in the vehicle, KF-1X, KF-2X, and KF-5X groups was dominated by the phyla *Firmicutes* (65% for the vehicle group, 69% for the KF-1X group, 51% for the KF-2X group, and 57% for the KF-5X group) and *Bacteroidetes* (28% for the vehicle group, 25% for the KF-1X group, 43% for the KF-2X group, and 39% for the KF-5X group). The gut microbiotas of the mice were dominated by *Firmicutes* and *Bacteroidetes* (together accounting for approximately 90%). However, KF-2X- and KF-5X-treated mice had a reduced proportion of *Firmicutes* and an increased proportion of *Bacteroidetes*. The *Firmicutes*/*Bacteroidetes* (F/B) ratios in the KF-5X and KF-2X groups were 1.46 and 1.19, respectively, which were lower than those in the KF-1X (2.76) and vehicle (2.32) groups. The distinct gut microbiota compositions in the vehicle group were compared with those of the KF-1X (Figure 7c), KF-2X (Figure 7d), and KF-5X (Figure 7e) groups. As shown in Figure 7c, the linear discriminant analysis effect size (LEfSe) indicated that number of bacteria from the family *Ruminococcaceae* was higher in the KF-1X group than in the vehicle group. Figure 7d shows that the proportion of *Bacteroidales* and *Bacteroidia* was higher in the KF-2X group than in the vehicle group, whereas the proportion of *Clostridiales* and *Clostridia* was higher in the vehicle group than in the KF-2X group. Figure 7e shows that the KF-5X-treated mice had higher proportions of *Rikenellaceae*, *Bacteroidales*, and *Bacteroidia* than did the mice from the vehicle group. However, mice from the vehicle group had higher proportions of *Clostridia* than did the KF-5X-treated mice.

## 4. Discussion

Probiotics naturally occur in fermented foods such as yogurt, sauerkraut, cabbage kimchee, and soy miso and natto [26]. Probiotics can have a positive effect on athletic performance by enhancing fatigue recovery, improving immune function, and maintaining healthy gastrointestinal function [26]. In addition, certain probiotics, such as *Bacillus coagulans*, have been shown to increase nutrient absorption. They are especially effective in protein absorption, particularly the absorption of leucine from whey protein [27]. In this study, we found KF may improve endurance performance without training by increasing probiotics or protein utilization.

Grip strength must be improved through procedural exercise training [28]. As shown in Figure 3, the grip strength in the KF-1X, KF-2X, and KF-5X groups significantly increased by 23%, 25%, and 26%, respectively, compared with that in the vehicle group. Thus, KF increased the grip strength of the test animals without providing training interventions. Therefore, long-term KF supplementation can benefit muscle strength even when exercise training is not implemented. Esgalhado et al. [29] and LeBlanc et al. [30] pointed out that probiotic or prebiotic supplementation could increase intestinal short-chain fatty acid (SCFA) content. These SCFAs affect lipid, glucose, and cholesterol metabolism and maintain intestinal integrity [31]. SCFAs, such as *n*-butyrate, acetate, and propionate, can regulate host energy balance and increase nutrient availability [32]. On the other hand, KF grains contain lactic acid bacteria and yeasts [33] and lactic acid bacteria have beneficial effects on the host [20]. Chen et al. [34] reported that *L. plantarum* TWK10 (LP10) increases muscle mass and grip strength, enhances exercise performance, and decreases physical fatigue. *Lactobacillus* spp. may influence exercise performance by producing lactate. However, lactate-utilizing bacteria can use lactate to produce butyrate [35]. In addition, a significant amount of blood lactate is produced after acute exercise [36]. Thus, KF may use lactate to produce SCFAs and then increase nutrient availability and improve exercise performance. Therefore, KF may be a potential solution for the removal and utilization of blood lactate and, thus, reduce physical fatigue.

The accumulation of ammonia in the blood and brain during exercise has a negative effect on the central nervous system and leads to fatigue [37]. Therefore, KF supplementation may play an important role in the relationship between the central nervous system and fatigue by decreasing the accumulation of blood ammonia. However, no significant differences were observed in the blood glucose levels among the vehicle, KF-1X, KF-2X, and KF-5X groups after the 10-min swimming test or after the 10-min swimming test and 20-min rest period (Figure 3e,f). The increased BUN level reflects protein decomposition, which has an adverse effect on the muscle contraction strength and leads to fatigue. Therefore, BUN is an important biochemical parameter related to fatigue [38,39,40]. The result indicated that KF supplementation can decrease BUN levels, thereby reducing the fatigue induced by acute exercise. Serum CK levels are important clinical biomarkers for muscle damage, muscular dystrophy, and severe muscle breakdown [41]. Muscle cell damage leads to the accumulation of metabolites during exercise, resulting in decreased exercise capacity [42]. Therefore, KF supplementation can decrease the serum CK level and ameliorate skeletal muscle injury induced by acute exercise. Chen et al. [34] demonstrated that LP10 supplementation triggers an anti-fatigue effect by lowering serum lactate, ammonia, and CK levels, thereby enhancing exercise performance in mice. Glucose is stored as glycogen, which is mainly present in the liver and muscle tissues. Glycogen content is a determining factor for fatigue [43]. Muscle glycogen content is a limiting factor for prolonged exercise. KF can increase glycogen levels, which is beneficial for enhancing physical endurance. Therefore, KF-1X, KF-2X, and KF-5X groups can all increase liver or muscle glycogen contents, which may directly increase exercise performance and reduce physical fatigue. However, KF-5X can significantly increase muscle glycogen content than KF-1X and KF-2X.

The F/B ratios in the KF-5X and KF-2X groups were lower than vehicle group. In particular, *Bacteroidetes* is associated with increased expression of proteins involved in the catabolism of branched-chain amino acids and increased production of SCFAs [44]. Although SCFAs are a source of host calories, their intestinal production is mainly related to reduced inflammation, increased satiety, and overall positive metabolic effects [45,46]. Bomhof et al. [47] demonstrated that prebiotics and probiotics all reduce the F/B ratio. Other phyla of all groups were also detected, including the *Proteobacteria*, *Deferribacteres*, *Tenericutes*, *Cyanobacteria*, *Actinobacteria*, *Acidobacteria*, *Synergistetes*, and *TM7. Ruminococcaceae*, the most abundant bacterial family from the order *Clostridiales*, is found in the mammalian gut environment, and is associated with the maintenance of gut health [48]. The order *Bacteroidales* is known to provide beneficial properties to the host [49,50,51]. These results indicated that KF can modify the gut microbiota, thereby contributing to the metabolic networks that reduce physical fatigue.

## 5. Conclusions

In the present study, we found that four weeks of KF supplementation provided the potential to modulate the gut microbiota and yielded anti-fatigue activity by lowering plasma lactate, ammonia, and CK levels, thereby increasing the exercise performance and improving the forelimb grip strength and the swimming time to exhaustion in mice. In addition, KF may modify gut microbiota, thereby contributing to the host metabolic phenotype, which improves exercise performance and reduces physical fatigue. Therefore, KF might be useful in reducing physical fatigue. In the future, the molecular mechanism and clinical trial of KF involved in anti-fatigue should be investigated.

## Figures and Tables

**Table 1:** General characteristics of mice with kefir (KF) supplementation.

Characteristic	Vehicle	KF-1X	KF-2X	KF-5X	Trend Analysis
Initial BW (g)	33.4 ± 0.7	33.7 ± 0.7	33.4 ± 0.9	33.6 ± 0.3	0.4631
Final BW (g)	36.2 ± 2.2	36.7 ± 1.1	36.5 ± 1.4	36.0 ± 1.3	0.6215
Food intake (g/mouse/day)	6.2 ± 0.6	6.2 ± 0.3	6.1 ± 0.3	6.0 ± 0.3	0.1216
Water intake (mL/mouse/day)	9.5 ± 2.0	9.4 ± 2.0	9.5 ± 0.8	9.5 ± 2.5	0.8155
Liver (g)	2.03 ± 0.10	2.00 ± 0.26	2.06 ± 0.13	2.03 ± 0.13	0.7996
Kidney (g)	0.52 ± 0.03	0.53 ± 0.03	0.52 ± 0.05	0.51 ± 0.04	0.4781
EFP (g)	0.37 ± 0.03	0.35 ± 0.06	0.32 ± 0.03	0.32 ± 0.11	0.1673
Heart (g)	0.21 ± 0.02	0.23 ± 0.02	0.21 ± 0.02	0.22 ± 0.02	0.8400
Lung (g)	0.23 ± 0.03	0.22 ± 0.01	0.22 ± 0.02	0.22 ± 0.02	0.2464
Muscle (g)	0.36 ± 0.03 ^a^	0.39 ± 0.02 ^b^	0.39 ± 0.02 ^b^	0.39 ± 0.02 ^b^	0.0871
BAT (g)	0.09 ± 0.02 ^a^	0.12 ± 0.02 ^b^	0.11 ± 0.02 ^b^	0.11 ± 0.01 ^b^	0.1560
Relative liver weight (%)	5.60 ± 0.30	5.45 ± 0.72	5.65 ± 0.45	5.65 ± 0.35	0.3092
Relative kidney weight (%)	1.44 ± 0.09	1.45 ± 0.11	1.42 ± 0.14	1.42 ± 0.11	0.8171
Relative EFP weight (%)	1.01 ± 0.11	0.95 ± 0.16	0.87 ± 0.12	0.89 ± 0.32	0.1797
Relative heart weight (%)	0.59 ± 0.08	0.62 ± 0.07	0.58 ± 0.06	0.62 ± 0.07	0.6835
Relative lung weight (%)	0.62 ± 0.07	0.59 ± 0.03	0.61 ± 0.04	0.61 ± 0.05	0.2464
Relative muscle weight (%)	0.99 ± 0.15 ^a^	1.07 ± 0.04 ^ab^	1.07 ± 0.06 ^ab^	1.09 ± 0.05 ^b^	0.0899
Relative BAT weight (%)	0.24 ± 0.06 ^a^	0.33 ± 0.05 ^b^	0.31 ± 0.05 ^b^	0.30 ± 0.04 ^b^	0.1607

Data are presented as mean ± SD, *n* = 8 mice/group. Different letters (^a^, ^b^) in the same row indicate a significant difference at *p* < 0.05. Muscle mass includes both gastrocnemius and soleus muscles in the back part of the lower legs. BW, body weight; BAT, brown adipose tissue; EFP, epididymal fat pad. The mice in the vehicle, KF-1X, KF-2X, and KF -5X groups were pretreated for four weeks.

**Table 2:** Biochemical analysis with kefir (KF) supplementation at the end of the experiment.

Parameter	Vehicle	KF-1X	KF-2X	KF-5X	Trend Analysis
AST (U/L)	62 ± 13	53 ± 13	52 ± 10	52 ± 13	0.1562
ALT (U/L)	45 ± 19 ^b^	37 ± 12 ^ab^	30 ± 6 ^a^	30 ± 8 ^a^	0.0290
Albumin (g/dL)	3.3 ± 0.1	3.3 ± 0.1	3.3 ± 0.1	3.4 ± 0.1	0.0437
Creatinine (mg/dL)	0.36 ± 0.07	0.35 ± 0.03	0.33 ± 0.02	0.34 ± 0.03	0.9029
LDH (mg/dL)	268 ± 95	250 ± 78	246 ± 20	244 ± 75	0.6979
CK (U/L)	392 ± 106 ^b^	298 ± 73 ^a^	271 ± 54 ^a^	276 ± 88 ^a^	0.0063
TP (g/dL)	4.41 ± 0.17	4.45 ± 0.05	4.51 ± 0.06	4.50 ± 0.09	0.0914
Glucose (mg/dL)	79 ± 14	80 ± 4	75 ± 7	80 ± 8	0.5622
TC (mg/dL)	134 ± 16	134 ± 10	121 ± 8	122 ± 13	0.0228
TG (mg/dL)	168 ± 23	163 ± 20	160 ± 23	162 ± 34	0.8584

Data are presented as mean ± SD, *n* = 8 mice/group. Different letters (**^a^**, **^b^**) in the same row indicate a significant difference at *p* < 0.05 determined through one-way ANOVA. Vehicle (glucose water), KF-1X (2.15 g/kg/day KF), KF-2X (4.31 g/kg/day KF), and KF-5X (10.76 g/kg/day KF). AST, aspartate aminotransferase; ALT, alanine aminotransferase; LDH, lactate dehydrogenase; CK, creatine kinase; TP, total protein; TC, total cholesterol; TG, triacylglycerol.
